# Circularly polarized luminescence from supramolecular assemblies based on small molecules

**DOI:** 10.1002/smo.20240061

**Published:** 2025-05-30

**Authors:** Xinyu Liu, Xiaoyan Wang, Xiaotao Zhang, Liqiang Li, Yu Wang, Wenping Hu

**Affiliations:** ^1^ Department of Chemistry School of Science Key Laboratory of Organic Integrated Circuit Ministry of Education & Tianjin Key Laboratory of Molecular Optoelectronic Sciences Tianjin University Tianjin China; ^2^ Haihe Laboratory of Sustainable Chemical Transformations Tianjin China; ^3^ Joint School of National University of Singapore and Tianjin University International Campus of Tianjin University Fuzhou China

**Keywords:** chiral supramolecular assemblies, chiroptical materials, circularly polarized luminescence, organic small molecules, supramolecular chirality

## Abstract

Circularly polarized luminescence (CPL)‐active materials have a wide range of technological applications. Traditionally, creating CPL‐active materials relies on the use of chiral luminophores. In contrast, supramolecular assembly introduces an innovative and promising strategy for developing CPL‐active materials not only from chiral luminophores but also from achiral species. This approach significantly enriches the diversity of CPL‐active materials. It also offers an effective means to optimize the performance of CPL‐active materials, such as enhancing the asymmetry factor |*g*_lum_|. Compared to polymers, the assembly of small molecules is generally easier to control. This review systematically summarizes the recent progress and developments in CPL from small‐molecule assemblies, particularly focusing on differences, merits, and demerits of three typical assembly modes. The aim is to provide valuable insights for the future development of chiroptical materials.

## Graphical abstract

This minireview outlines the recent advances of circularly polarized luminescence (CPL)‐active supramolecular assemblies based on small molecules. It covers fundamental concepts of CPL, typical design strategies of CPL‐active materials—including the assembly of chiral luminophores, the assembly of chiral components and achiral luminophores, and the assembly of achiral luminophores—and their applications. Additionally, it discusses the challenges of this field.

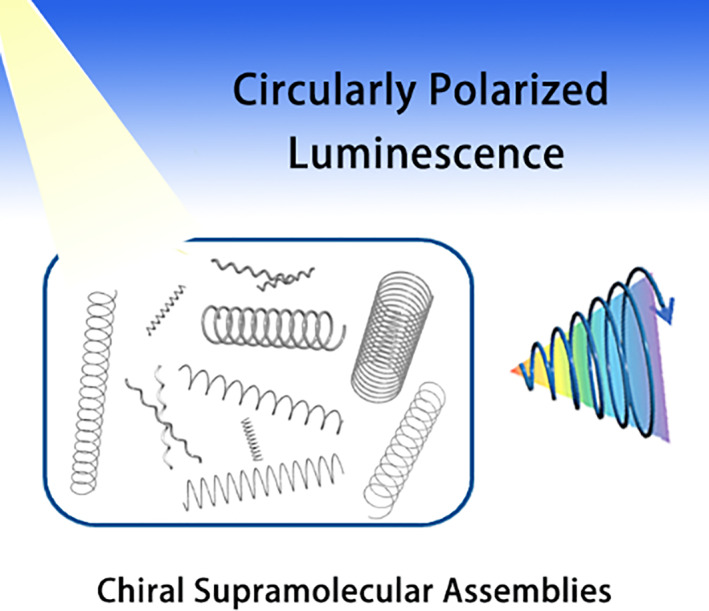

## 1 INTRODUCTION

The polarization of light refers to the distinction between the vibration direction and the propagation direction of the light wave. Light can be categorized as linearly, elliptically, partially, and circularly polarized light based on its polarization characteristics. Among these types, circularly polarized light is particularly unique, and has garnered considerable attention owing to its promising applications in three‐dimensional (3D) display technologies,^[1]^ optical sensing devices,[^2^, ^3^] photoelectric components,^[4]^ supramolecular chirality modulation,[^5^, ^6^] asymmetric synthesis,^[7]^ and so on.

In addition to employing the physical method, that is, utilizing a linear polarizer and a quarter‐wave plate, circularly polarized light can be inherently generated from chiral luminescent materials, thus avoiding potential energy loss associated with intermediate conversion steps. This approach generally requires chiral luminescent materials. For instance, the most common strategy for obtaining organic circularly polarized luminescence (CPL)‐active materials involves covalently linking a chiral moiety to a luminophore. However, this method often entails complex and time‐consuming synthesis processes, and the CPL activity of the resulting chiral emitters can be inherently unpredictable.^[8]^ Similarly, the fabrication of inorganic CPL‐active materials relies on intricate synthesis methods involving incorporating chiral ligands into prefabricated inorganic hosts, which is also challenging.[^9^, ^10^] Consequently, there is an urgent need to develop a general method for fabricating CPL‐active materials, addressing the limitations of current synthesis routes.

Supramolecular assembly^[11]^ offers a promising strategy for controlling the arrangements of building blocks and enhancing the inherent properties of materials in an effective manner. The generation of circularly polarized light from supramolecular assemblies requires the formation of chiral superstructures based on luminophores. When luminophores are situated in a dissymmetric environment upon photo‐ or electro‐excitation, CPL is produced. This assembly process offers a versatile platform to induce CPL activity in both chiral molecules and achiral substances, thereby expanding the realm of potential luminescent entities.[^12^, ^13^] Additionally, we can significantly improve the circular polarization properties of certain CPL‐active materials through supramolecular assembly. This efficient method is also appropriate for introducing a variety of organic and inorganic components into chiral superstructures, generating an extensive diversity of CPL‐active materials. Compared to polymer assemblies, the assembly of small molecules is more precise, primarily due to the more explicit and predictable nature of intermolecular interactions.^[14]^ This feature facilitates the systematic design and construction of supramolecular assemblies with tailored properties by meticulously selecting diverse small molecule components. Thus, the assembly of small molecules presents a unique strategy for creating customized and highly CPL‐active materials.

Currently, although several reviews about supramolecular‐assembly‐based CPL materials have been published, the majority of them focus on the molecular design and CPL amplification.[^12^, ^13^, ^14^, ^15^] The assembly modes were briefly mentioned. Besides, most reviews emphasized the assembly of polymers, whereas small molecules, which have more precise structures and diverse assembly modes, were not discussed in detail. Moreover, the field has grown rapidly, and many new exciting phenomena have emerged, such as CPL from supramolecular assemblies based on macrocycles.

Thus, in this review, we provide a comprehensive and timely summary on recent progress and developments in CPL from small‐molecule assemblies, focusing on the differences, merits, and demerits of three typical assembly modes. This review begins by outlining the fundamental concepts of CPL followed by an exploration of supramolecular assembly modes based on small molecules for generating circularly polarized light. Then, we introduce advanced applications of CPL‐active materials, concluding with discussions on the challenges and perspectives in this emerging field. By presenting representative examples, we aim to provide the readers with comprehensive insights into the design strategies for CPL‐active materials and their applications.

## 2 BASIC CONCEPTS RELATED TO CPL

**FIGURE 1:**
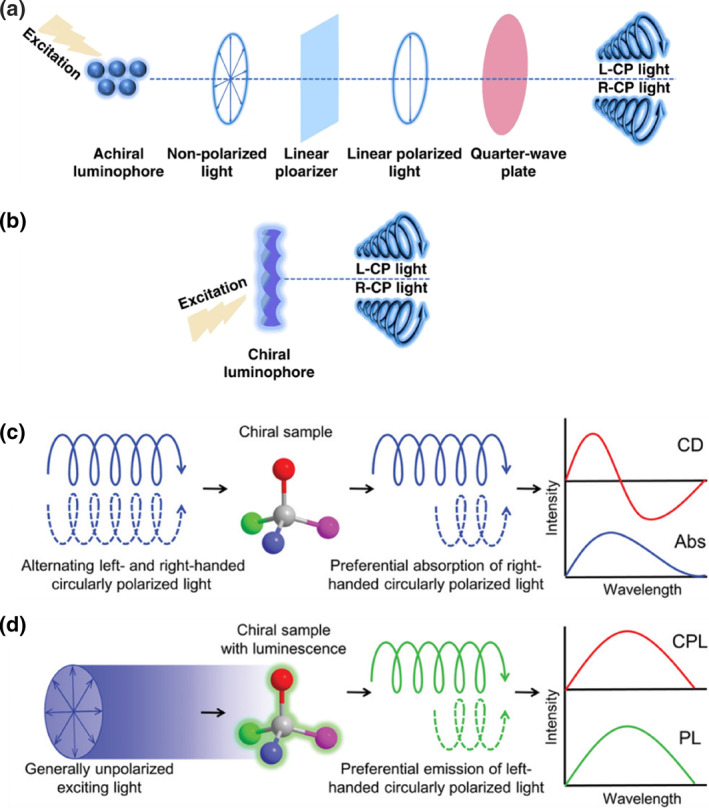
Methods for generating circularly polarized light. (a) Physical method. (b) Circularly polarized luminescence. (a, b) Reproduced with permission.^[15]^ Copyright 2021, Springer Nature. (c) Schematic illustration of circular dichroism (CD). (d) Schematic illustration of circularly polarized luminescence. (c, d) Reproduced with permission.^[12]^ Copyright 2019, Wiley‐VCH.

Circularly polarized light can be categorized by its handedness: left‐handed circularly polarized (L‐CP) light rotates clockwise (CW), while right‐handed circularly polarized (R‐CP) light rotates counterclockwise (CCW). Traditional methods for generating circularly polarized light typically involve a two‐step physical process: first, converting unpolarized light into linearly polarized light utilizing a linear polarizer, and then passing this linearly polarized light through a quarter‐wave plate (Figure 1a).^[15]^ In addition to the physical approach, circularly polarized light can be directly generated from the luminescence of chiral luminophores, a phenomenon known as CPL (Figure 1b).^[15]^ CPL exhibits different emission intensity of L‐CP and R‐CP light in chiral luminescent systems under photo‐ or electroexcitation.

As mentioned above, the direct generation of circularly polarized light is related to chiral emissive materials. To investigate the photophysical properties of chiral emissive materials, electronic circular dichroism (CD) and CPL spectroscopy are commonly employed. CD spectroscopy characterizes the ground‐state electronic features of chiral substances by measuring their differential absorption of L‐CP and R‐CP light. The CD spectrum is derived from comparing the original and remaining light intensities, with the Cotton effect typically observable at the absorption peaks. In contrast, CPL spectroscopy directly demonstrates the ability of chiral systems to spontaneously emit either L‐CP or R‐CP light, typically representing the excited‐state properties of chiral materials.[^16^, ^17^] It is noteworthy that the presence of a CD signal in a chiral luminescent system does not guarantee the observation of a CPL signal, because they are related to the ground‐ and excited‐state properties of materials, respectively. On the contrary, CPL‐active materials typically exhibit the Cotton effect.

As depicted in Figure 1c,d, both CD and CPL spectroscopy rely on the intensity of circularly polarized light, thereby rendering the quantification of CD and CPL comparable in their magnitude assessment.^[12]^ Specifically, the extent of CD can be quantitatively characterized by the absorption dissymmetry factor as *g*_abs_ (or *g*_CD_), which is defined by Equation (1):

(1) $$g_{\text{abs}}=\frac{\varepsilon_{L}-{\;\varepsilon }_{R}}{1/2\left(\varepsilon_{L}+\varepsilon_{R}\right)}=\frac{2\left(\varepsilon_{L}-{\;\varepsilon }_{R}\right)}{\varepsilon_{L}+{\;\varepsilon }_{R}}$$

where *ε*_L_ and *ε*_R_ are the molar absorption coefficients of L‐CP and R‐CP light, respectively. The *g*_abs_ value can be experimentally determined by Equation (2):

(2) $$g_{\text{abs}}=\frac{\text{ellipticity}}{32980\times \text{absorbance}}$$

where the “ellipticity (in medg)” and “absorbance” values can be directly obtained from the CD spectra.

Similarly, the CPL property can be evaluated by the luminescence dissymmetry factor *g*_lum_ (*g*_PL_ and *g*_EL_ are also used to differentiate the *g*_lum_ value of photoluminescence and electroluminescence) described by Equation (3):^[18]^

(3) $$g_{\text{lum}}=\frac{I_{L}-{\;I}_{R}}{1/2\left(I_{L}+I_{R}\right)}=\frac{2\left(I_{L}-{\;I}_{R}\right)}{I_{L}+I_{R}}$$

where *I*_L_ and *I*_R_ represent the intensities of L‐CP light and R‐CP light, respectively. According to this formula, a *g*_lum_ value of 0 indicates unpolarized light, while values of ±2 signify purely left‐ or R‐CP light.

Furthermore, studies have shown that the magnitude of *g*_lum_ is influenced by transition probabilities (Fermi's Golden Rule) (Equation 4)^[18]^:

(4) $$g_{\text{lum}}=4\;\cos\;\theta \frac{\left|m\right|\left|\mu \right|}{{\left|m\right|}^{2}+{\left|\mu \right|}^{2}}\approx 4\;\cos\;\theta \frac{\left|m\right|}{\left|\mu \right|}$$

where *μ* and *m* represent the electric and magnetic dipole transition moment, respectively, and *θ* is the angle between them. In most molecular systems, it is a well‐established fact that *m* is substantially smaller than *μ*. Thus, achieving a large *g*_lum_ value typically requires the system to permit magnetic dipole transitions while prohibiting electric dipole transitions. In other words, systems with relatively small *|μ|* tend to exhibit higher *g*_lum_ values.

Additionally, the quantum yield (Φ) is a crucial metric for evaluating CPL‐active materials, defined by Equation (5):

(5) $$\Phi =\frac{\text{number}\;\text{of}\;\text{photons}\;\text{emitted}}{\text{number}\;\text{of}\;\text{photons}\;\text{absorbed}}$$

the value of Φ ranges from 0% to 100%. For instance, if a system absorbs 100 photons and emits 30, its quantum yield will be 30%.

## 3 CPL‐ACTIVE SUPRAMOLECULAR ASSEMBLIES

**FIGURE 2:**
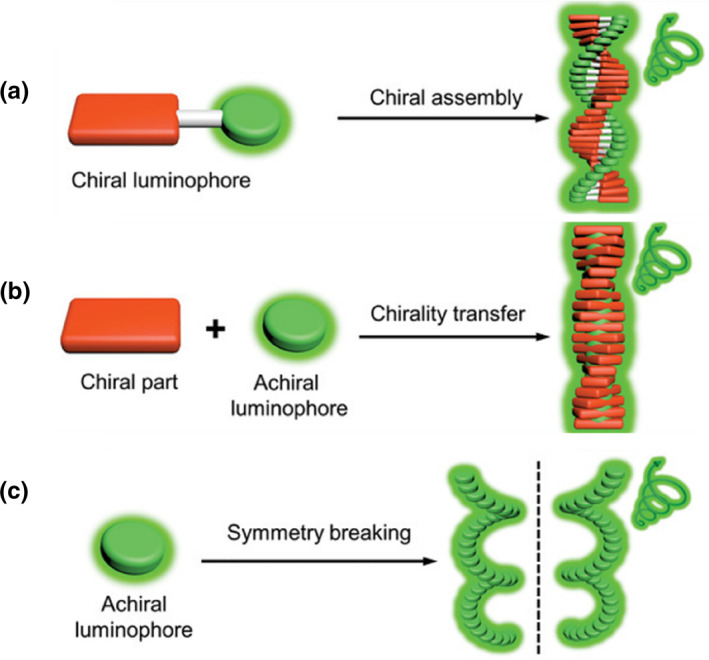
Three typical assembly modes for fabricating CPL‐active supramolecular assemblies. (a) The assembly of chiral luminophores. (b) The assembly of chiral components with achiral luminophores. (c) The assembly of achiral luminophores. (a–c) Reproduced with permission.^[12]^ Copyright 2019, Wiley‐VCH.

**FIGURE 3:**
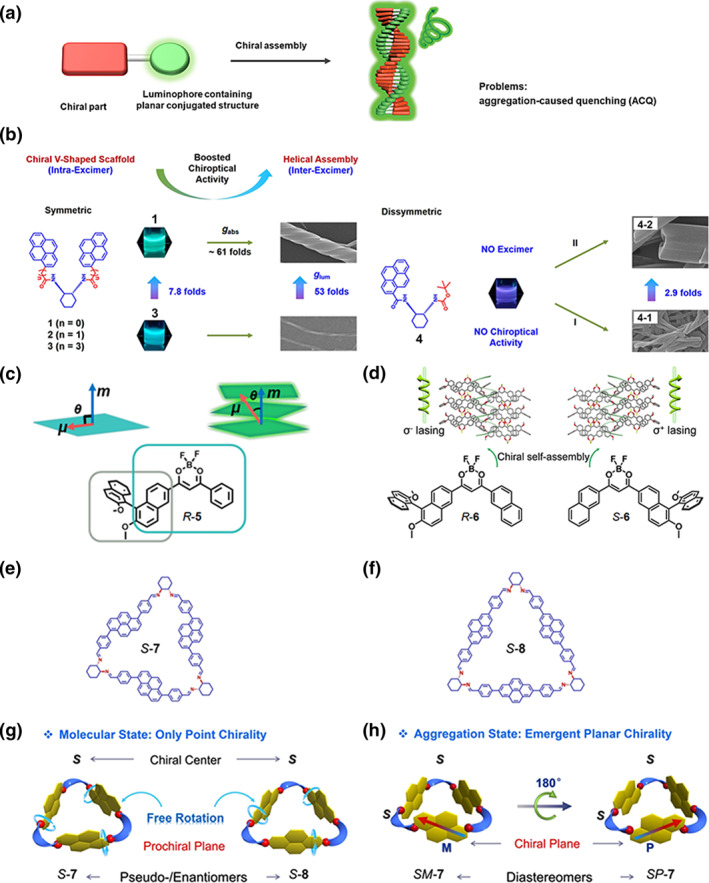
(a) CPL is generated from helical assemblies of chiral luminescent molecules based on luminophores containing planar conjugated structures. (b) Amplification of chiroptical properties from supramolecular assemblies based on chiral V‐shaped pyrene derivatives (**1–4**): the assemblies of symmetric molecules (**1–3**) showed spacer‐dependent CPL performance, while the assemblies of dissymmetric molecule **4** displayed pathway‐dependent CPL properties. Reproduced with permission.^[22]^ Copyright 2021, Wiley‐VCH. (c) Molecular structure of *R*‐**5** and schematic illustration of *μ* and *m* within planar chromophores and chiral stacking involving electronic coupling. Reproduced with permission.^[23]^ Copyright 2024, Wiley‐VCH. (d) Molecular structures of **6** and the chiral superstructures induced by the chiral binaphthol skeletons. Reproduced with permission.^[24]^ Copyright 2024, Wiley‐VCH. (e, f) Molecular structures of the chiral triangles (e) *S‐* **7** and (f) *S* **‐8**. (g, h) Illustration of the macrocyclic conformers at the (g) molecular and (h) aggregation state. only the *S*‐enantiomers were shown for clarity. (e–h) Reproduced with permission.^[25]^ Copyright 2022, Wiley‐VCH. CPL, circularly polarized luminescence.

Chirality and luminescence are prerequisites for fabricating CPL‐active materials.^[19]^ For an assembled system, the chiral information can be achieved through the asymmetric arrangement of the building blocks.^[20]^ In this regard, both chiral and achiral luminescent molecules can potentially generate CPL. This section summarizes three typical assembly modes for fabricating CPL‐active supramolecular assemblies: (1) the assembly of chiral luminophores (Figure 2a); (2) the assembly of chiral components with achiral luminophores (Figure 2b); (3) the assembly of achiral luminophores (Figure 2c).

### 3.1 CPL from the assembly of chiral luminescent molecules

**FIGURE 4:**
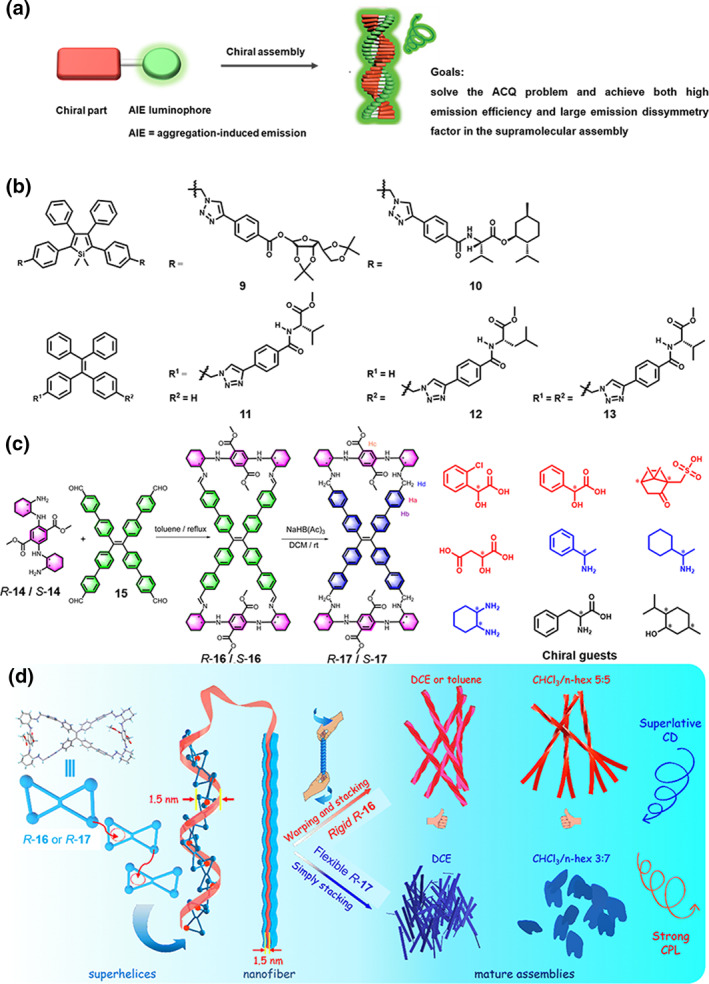
(a) CPL is generated from helical assemblies of chiral luminescent molecules bearing AIE luminophores. (b) Examples of AIE systems based on non‐cyclic molecules with CPL originated from helical aggregation. (c) Synthetic routes of the TPE bimacrocycles **16**/**17** and molecular structures of the chiral recognition molecules. (d) Schematic illustration of the assembly process of *R*‐**16** and *R*‐**17.** (c, d) Reproduced with permission.^[27]^ Copyright 2024, Royal Society of Chemistry. CPL, circularly polarized luminescence.

The exhibition of CPL activity in molecules that incorporate both luminescent and chiral moieties is not guaranteed, particularly when the chiral center is positioned adjacent to a luminescent unit or when the chiral molecule possesses a flexible conformation. The assembly of chiral luminescent molecules offers a promising avenue for constructing CPL‐active materials. Typically, these chiral luminescent molecules consist of a chiral moiety and a luminescent moiety connected by covalent bonds, enabling the entire molecular system to exhibit CPL characteristics, even if the chiral moiety itself is non‐luminescent.^[21]^ Integrating chiral and luminescent units into chiral luminescent molecules primarily encompasses two approaches: one is integrating luminophores containing planar conjugated structures with chiral moieties (Figure 3a)^[26]^; the other is coupling aggregation‐induced emission (AIE)‐active luminophores with chiral moieties (Figure 4a).^[26]^ Both strategies facilitate the formation of chiral supramolecular systems with CPL properties. Building upon these foundations, we review several representative examples.

#### 3.1.1 Chiral luminescent molecules based on luminophores containing planar conjugated structures

Liu et al. have designed a series of symmetric and asymmetric chiral V‐shaped pyrene derivatives linking achiral pyrene units with chiral *trans*‐1,2‐diaminocyclohexane scaffolds through various spacer groups. They explored circularly polarized excimer emission (CPEE) in their supramolecular assemblies in comparison to the molecular states (Figure 3b).[^22^, ^28^, ^29^, ^30^, ^31^] As depicted in Figure 3b, Liu et al. discovered that the two pyrene units are locked together in specifically designed symmetric molecules, promoting the formation of intramolecular excimers that displayed spacer‐dependent CD and CPEE.^[22]^ With increased molecular rigidity, the symmetric molecules (**1, 2, 3**) exhibited amplified chiroptical properties in the molecular state. Compared to **2** and **3**, **1** formed an unprecedented hexagonal superhelical structure, where the V‐shaped conformation aligns with the nanofiber architecture. The *g*_abs_ of the superhelical structure was 3.59 × 10^−2^, which value is approximately 60‐fold of that for **1**. The *g*_lum_ of this superhelical structure was 2.64 × 10^−2^, which is the highest reported for pyrene aggregates. In contrast, in the monomeric state of asymmetric molecule **4**, neither CD nor CPL signals were detected due to the absence of intramolecular excimers. Upon assembly, the asymmetric molecules **4** formed two distinct hexagonal aggregates controlled by supramolecular packing modes.^[22]^ The assemblies showed significantly amplified |*g*_lum_| owing to strong intermolecular excimers. Through the supramolecular assembly, not only was the internal rotation of the molecules constrained—reducing non‐radiative transitions—but efficient chirality transfer and excimer formation were also enhanced under either intra‐ or intermolecular confinement. All these factors contributed to the amplification of |*g*_lum_|.

Intermolecular electron coupling can also enhance |*g*_lum_|. For example, Yang et al. utilized highly emissive difluoroboron β‐diketonate dyes *R*‐**5** containing the chiral binaphthol skeletons to form microcrystals through supramolecular assembly (Figure 3c).^[23]^ The chiral microcrystals exhibited intense CPL signals with a |*g*_lum_| value reaching up to 0.11, far exceeding most reported organic CPL emission systems (Figure 3c). Through single‐crystal X‐ray diffraction (XRD) analysis and theoretical calculations, the study revealed the influence of intermolecular electron coupling in chiral supramolecular assemblies on the excited‐state electronic structure and electron transitions. This coupling increased the cos*θ*_*μ*,*m*_ value from 0.05 (monomer) to 0.86 (tetramer), thus triggering the strong optical activity of **5**. The research results indicated that by regulating the orientation and extent of intermolecular electron coupling, the optical activity of chromophores in the assemblies can be adjusted, providing new ideas for the design of novel chiral optical functional materials. Subsequently, using molecules **6** with similar building blocks, they achieved intrinsic circularly polarized lasing emission with high *g*_lum_ up to 1.0 (Figure 3d).^[24]^

Besides conventional non‐cyclic chiral building blocks, chiral macrocycles containing planar conjugated structures also provide a powerful new strategy to fabricate CPL materials. Liu et al. designed chiral triangles *S*‐**7** and *S*‐**8**, using *trans*‐1,2‐cyclohexanediamine with point chirality as vertices and achiral dialdehyde‐functionalized pyrenes as edges.^[25]^ *S‐* **7** has both configurational point chirality from the vertices and conformational planar chirality from the zigzag‐shaped pyrene edges (Figure 3e), while *S‐* **8** with linear pyrene edges lacks conformational chirality (Figure 3f). At the molecular state, the chiroptical properties of *S‐* **7** and *S‐* **8** are dominated by the point chirality due to the free rotation of the self‐isolated pyrene edge (Figure 3g). However, after supramolecular assembly, the free rotation of the edge is limited, thus planar chirality overwhelms point chirality. In different solvent polarity, *S‐* **7** forms nanotwists with opposite handedness (Figure 3h). In non‐polar media, *S*‐**7** generated negative CD and CPL signals. In polar media, the signs of CD and CPL were inverted. Beyond CD and CPL analyses, scanning electron microscopy and theoretical calculations have further validated that the overall chiral manifestation of the assemblies is predominantly governed by the newly emerged planar chirality.

#### 3.1.2 Chiral luminescent molecules based on AIE‐active luminophores

Molecules featuring planar conjugated luminophores and chiral moieties, driven by *π*‐*π* stacking interactions, can form helical assemblies that exhibit CPL properties. However, the resultant excimers or exciplexes often suffer from aggregation‐caused quenching (ACQ), significantly compromising the luminescence efficiency and spectral stability. To address the limitations posed by ACQ, innovative molecular design strategies are essential for the development of efficient luminescent materials. In this context, Tang's group^[32]^ observed an intriguing phenomenon: a propeller‐shaped *π*‐conjugated molecule, 1‐methyl‐1,2,3,4,5‐pentaphenylsilane, exhibits negligible luminescence in dilute solutions, but a Φ_F_ surges by over two orders of magnitude upon aggregation. They termed this phenomenon AIE, which ignited considerable interest in the interplay between AIE and chiral properties.

Building on this discovery, they pioneered the integration of AIE‐active fluorogens with chiral units to construct high‐performance CPL‐active materials in 2012 (Figure 4a). They designed molecule **9** containing the AIE‐active luminophore 1,1‐Dimethyl‐2,3,4,5‐Tetraphenylsilole (TPS) and chiral sugar moieties (Figure 4b).^[26]^ Experimental results demonstrated a remarkable 136‐fold enhancement in Φ_F_ from 0.6% in solution to 81.3% in the solid state, overcoming the limitation of ACQ associated with conventional luminescent materials. The restriction of low‐frequency intramolecular motions is responsible for the AIE effect according to time‐resolved fluorescence (FL) studies and first‐principle theoretical calculation. Moreover, the helical assemblies of **9** exhibited robust CPL with *g*_lum_ values ranging from 0.08 to 0.32 and exceptional spectral stability (remaining stable for over 6 months under ambient conditions).[^35^, ^36^] They subsequently designed and synthesized molecule **10**, an amino acid containing TPS, which exhibits chiral and AIE properties (Figure 4b).^[37]^ Similarly, integrating chiral amino acids into *π*‐conjugated systems with AIE effects has pronounced efficacy in fabricating CPL‐active materials with both high |*g*_lum_| and Φ_F_.

**FIGURE 5:**
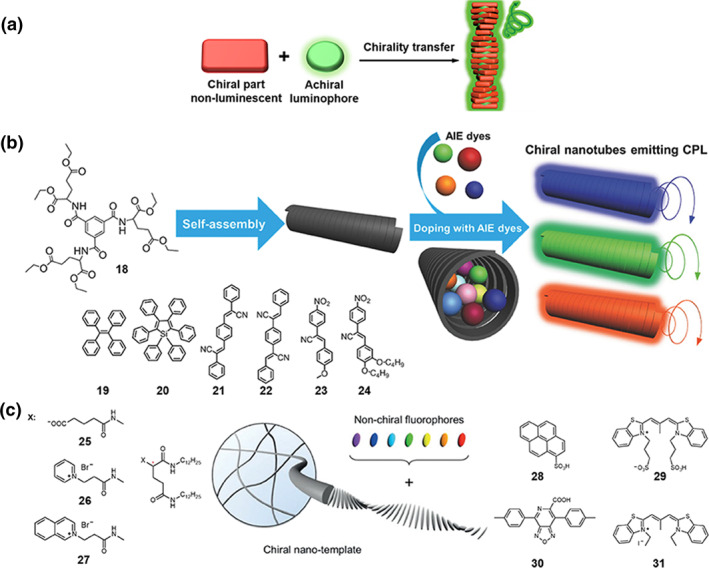
(a) The assembly of chiral non‐luminescent components with achiral luminophores. (b) Schematic illustration of CPL induced by encapsulating AIE dyes (**19–24**) within the hexagonal nanotube formed by the chiral gelator **18**. Reproduced with permission.^[33]^ Copyright 2017, Wiley‐VCH. (c) Schematic illustration of CPL generation by incorporating negatively charged achiral dyes (**28–31**) into nanotemplates based on positively charged *L*‐glutamic acid derivatives (**25–27**). Reproduced with permission.^[34]^ Copyright 2017, Wiley‐VCH. CPL, circularly polarized luminescence.

While TPS derivatives achieve both high Φ_F_ and |*g*_lum_| through supramolecular assembly, their applications are constrained by demanding reaction conditions and complex synthetic routes. To overcome these challenges, chemists have turned to the supramolecular assembly of chiral tetraphenylethene (TPE) derivatives.^[38]^ In 2014, Tang and Li et al.^[39]^ proposed a strategy leveraging the assembly of chiral AIE molecular TPE derivatives to construct high‐efficiency fluorescent nanofibers. By strategically introducing an *L*‐valine methyl ester moiety into the classic AIE molecule TPE, they successfully synthesized molecule **11** in a high yield through a straightforward copper‐catalyzed azide‐alkyne “click” reaction (Figure 4b).^[39]^ This advancement not only endowed TPE molecule **11** with CPL capability but also facilitated its assembly into helical aggregates upon evaporation of a 1:9 dichloroethane/hexane mixture (*c* = 0.1 mM). Despite compound **11** has a relatively low average *g*_lum_ (0.03 within the 400–600 nm range) compared to the previously mentioned TPS derivatives, its convenient synthesis, availability of amino acid derivatives, and ability to form CPL helical supramolecular assembly make it an attractive candidate for future research. To further validate the generality of this strategy, another TPE derivative **12** based on *L*‐leucine was synthesized in the same reaction (Figure 4b).^[40]^ This derivative was also capable of forming helical fibers under similar conditions. Notably, these helical fibers exhibited a more pronounced wavelength‐dependent *g*_lum_, with a peak intensity of 0.07 at 385 nm, which gradually declined to approximately 0.02 at 600 nm while maintaining comparable CPL signal intensity to *L*‐valine derivative **11**. Further exploration of molecule **13** (Figure 4b),^[41]^ which incorporates two valine attachments revealed an unexpected outcome. Despite the identical chirality of the amino acid derivatives, these fibers exhibited an opposite helical direction compared to the single‐valine‐linked derivative **11**.^[41]^ Moreover, the *g*_lum_ of **13** was merely −0.003, an order of magnitude lower in absolute value and with an opposite sign compared to TPE derivatives **11** containing a single valine due to the reversed helicity. In contrast, both monofunctionalized scaffolds **11** and **12** exhibited positive values. This finding underscores the profound impact of molecular structural complexity on helical chirality and CPL properties, offering fresh insights into the mechanism governing the assembly process and the optical properties of these architectures. In the realm of CPL‐active supramolecular assemblies, a diverse array of AIE systems has been harnessed, mainly encompassing various TPS^[36]^ and TPE^[42]^ derivatives as well as Schiff base moieties.^[43]^

In addition to enhancing the Φ_F_, TPE‐based molecular macrocycle assemblies also exhibit chiral recognition ability. Zheng et al. synthesized TPE imine bismacrocycles **16** through the condensation reaction of chiral diaminocyclohexyl‐*p*‐diaminoterephthalate **14** and TPE tetrabenzaldehyde **15**, and then reduced the imine bonds to obtain more stable TPE tetramine bimacrocycle **17** (Figure 4c).^[27]^ These compounds (*R‐* **16** */S*‐**16** and *R*‐**17**/*S*‐**17**) can form superhelices in the supramolecular assembly through self‐inclusion. They displayed a |*g*_lum_| value of up to 0.039 and CD signals exceeding 7000 mdeg. This is quite rare among common organic compounds (Figure 4d). Taking advantage of this strong CD effect, the chiral TPE bimacrocycles showed excellent recognition capability toward chiral acids. In addition, they can also recognize chiral amines, chiral amino acids, and even neutral chiral alcohols through host‐guest interactions (Figure 4c). Meanwhile, these strong CD signals were also used for the high accuracy determination of the enantiomeric purity of chiral amine and chiral acid compounds at low concentrations.

In summary, the presence of luminescent and chiral moieties does not automatically confer CPL activity, particularly when the chiral center is spatially proximal to the luminescent unit or when the chiral molecule possesses a flexible conformation. To address this limitation, the assembly of chiral luminophores offers a promising assembly mode for constructing CPL‐active materials. However, this assembly mode requires complex covalent synthesis methodologies to effectively combine the chiral units with achiral luminophores, posing a significant challenge. Moreover, the optimization of CPL is intricately linked to various factors, including the spacer connecting the chiral center to the assembly site, the strength of the non‐covalent interactions, and the interplay between chiral and achiral moieties.

### 3.2 CPL from the assembly of chiral molecules and achiral luminescent molecules

**FIGURE 6:**
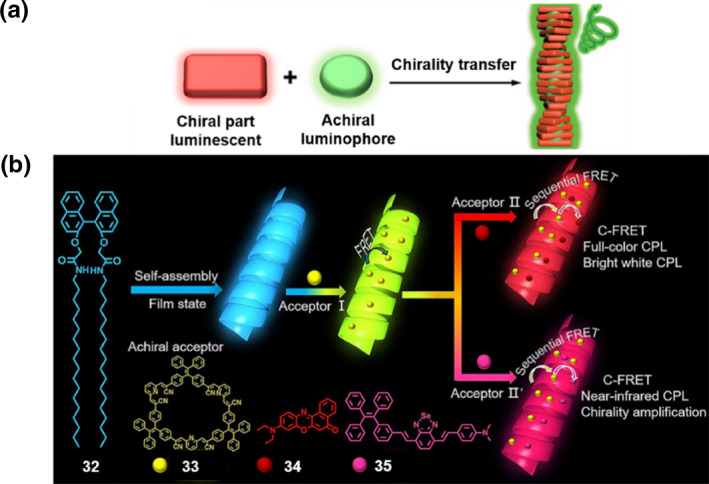
(a) The assembly of chiral luminescent components with achiral luminophores. (b) Schematic illustration of the process of chiral light‐harvesting and full‐color circularly polarized luminescence. In this system, **32** functioned as an initiator of chirality, **33** served as a conveyor, **34–35** were the terminal acceptors. Reproduced with permission.^[44]^ Copyright 2022, American Chemical Society.

Another effective assembly mode for fabricating CPL‐active materials is the combination of chiral molecules with achiral luminescent molecules through non‐covalent interactions. Two typical strategies are summarized based on whether chiral molecules are luminescent or not: (1) the assembly of chiral non‐luminescent molecules and achiral luminescent molecules (Figure 5a); (2) the assembly of chiral luminescent molecules and achiral luminescent molecules (Figure 6a). Considering that the luminophores utilized remain non‐chiral, the generation of CPL can be attributed to the induced chirality through the supramolecular assembly of achiral luminophores with chiral matrices, which has been extensively demonstrated in previous works and reviews.[^11^, ^45^, ^46^, ^47^] Chirality transfer refers to the transfer of chiral information from chiral components to the supramolecular assemblies.

#### 3.2.1 Chiral information from chiral non‐luminescent molecules

Chirality can be conferred through confinement within chiral environments. Chemists constructed chiral micro/nanostructures as chiral templates to encapsulate achiral luminophores, thereby inducing CPL.[^48^, ^49^, ^50^] For instance, chiral gelators that spontaneously form supramolecular gels with micro‐ or nanostructures serve as advantageous templates. A general approach has been developed to fabricate CPL‐active materials by the adaptive and cooperative accumulation of various achiral dyes in supramolecular nanotubes. Liu et al. found that a chiral gelator **18** can form chiral nanotubes due to the presence of three amide groups connected to the phenyl core, which gives stable hydrogen bond networks (Figure 5b).[^33^, ^51^] Then, the achiral AIE luminophores (**19–24**) were encapsulated into these confined chiral nanotubes via organogelation. These assemblies with AIE luminophores can emit CPL upon excitation.^[33]^

Besides the chiral encapsulation effect, electrostatic interactions between negatively charged achiral dyes and positively charged chiral nanotemplates can also be harnessed to induce robust and tunable CPL. In 2017, Ihara et al.^[34]^ utilized positively charged *L*‐glutamic acid derivatives (**25–27**) to construct chiral ordered nanotemplates (Figure 5c) via hydrogen bonding interactions between amide bonds.[^34^, ^52^] These templates effectively interacted with negatively charged achiral dyes (**28–31**), leading to the formation of panchromatic CPL‐active aggregates with H‐stacking features. The enhanced chirality of the templates and the electrostatic interactions between the dyes and the templates led to a remarkably intense CPL activity, with |*g*_lum_| value reached as high as 0.102 in the binary system of the cationic **29** with an anionic **26** derivative. Additionally, a remarkable enhancement in Φ_F_ was observed in the mixed system, surpassing the achiral dye (e.g., **29** with Φ_F_ = 0.16%) by up to 47‐fold (Φ_F_ = 7.5%).^[34]^ This FL enhancement was attributed to the chiral nanotemplates creating a microenvironment with high rotational resistance for dye molecules, effectively suppressing non‐radiative transitions and enhancing radiative emission efficiency. Careful selection of dyes allowed for tunable emission bands, enabling the assembly of diverse color‐tunable CPL‐active materials.^[34]^

#### 3.2.2 Chiral information from chiral luminescent molecules

Zang et al. constructed a chiral light‐harvesting system (C‐LHS) with a sequential circularly polarized FL resonance energy transfer (C‐FRET) by combining the chirality and light‐absorbance initiator **32** with TPE macrocycles **33** featuring AIE and red/near‐infrared emissive dye molecules **34–35** (Figure 6b).^[44]^ This achievement enabled continuous chirality transmission/amplification and energy transfer. In this work, a pair of chiral supramolecular synthons **32** capable of self‐assembling into helical nanostructures was selected. Through supramolecular assembly, three energy acceptors **33–35** were successfully incorporated into the helical structures, yielding a C‐LHS with tunable FL colors for sequential energy and chiral transfer. The *g*_lum_ could reach up to 0.035. Research indicated that the close contact between donors and acceptors and their large spectral overlap are beneficial for energy transfer. Additionally, the rigid cavity structure of the green‐light‐emitting AIE macrocycle **33** and the stepwise host‐guest interactions allow the chirality of the supramolecular synthons **32** to be continuously and even amplified transferred to achiral dye molecules **34–35**. By adjusting the ratios among the components of the C‐LHS, the authors obtained an energy transfer efficiency as high as 98.5%, a Φ_F_ of 37%, a *g*_lum_ of 0.035, CPL with tunable emission colors, and CPL close to CIE standard white light over a wide wavelength range (360–800 nm). This work opens a new avenue for promoting efficient chiral transmission/amplification and mimicking natural white light.

**FIGURE 7:**
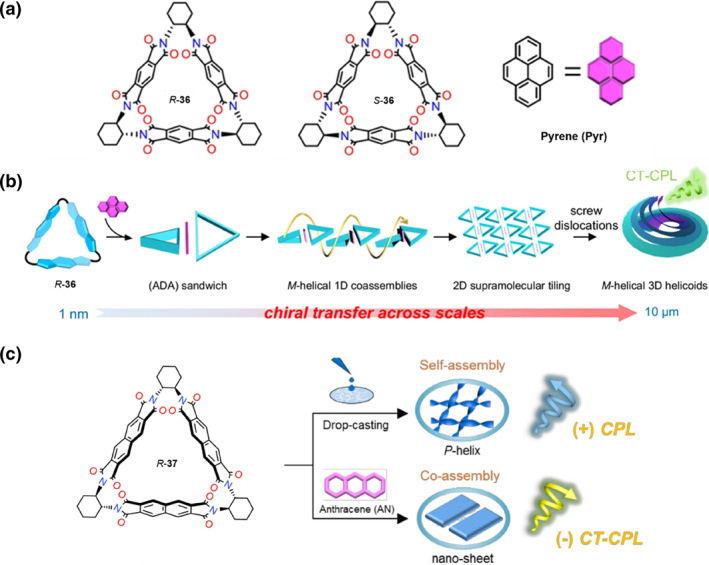
(a) Molecular structures of *R*‐**36**, *S*‐**36** and Pyrene, respectively. (b) The growth process of 3D helicoids featuring circularly polarized luminescence. (a, b) Reproduced with permission.^[53]^ Copyright 2024, Springer Nature. (c) Molecular Structure of *R*‐**37** and schematic illustration of *R*‐**37** self‐assembly and co‐assembly with anthracene. Reproduced with permission.^[54]^ Copyright 2025, Wiley‐VCH.

Macrocycles can also function as an initiator of chirality. Based on screw dislocations, Liu et al. proposed a hierarchical self‐assembly strategy to fabricate a macroscopic 3D helicoid featuring strong CPL.^[53]^ They selected the electron‐deficient chiral pyromellitic diimide‐based molecular triangle **36** as the electron acceptor and the size‐matched electron‐rich pyrene as the electron donor (Figure 7a). Through *π‐π* stacking and charge‐transfer (CT) interactions, pyrene was sandwiched between adjacent compound **36** molecules, leading to helical twists. Owing to the helical growth around screw dislocations, the CT complexes developed into curved microsheet subunits. The layer‐by‐layer screw dislocations promoted the formation of macroscopic homochiral helicoids through efficient chirality transfer (Figure 7b). These helicoids exhibited remarkable macroscopic helicity controlled by the molecular chirality of **36** and demonstrated excellent CPL properties with large |*g*_lum_| values up to 0.05. This work demonstrated the transfer and expression of chirality at the molecular, supramolecular, and macroscopic levels. In 2024, they designed a novel chiral 2,3,6,7‐naphthalenediimide‐based triangular macrocycle **37** with longer aromatic tetracarboxylic diimide linkers than **36**, and investigated its assembly modes (Figure 7c).^[54]^ Compared to **36**, which exhibits almost no emission in the solid state, **37** shows intense FL in both solution and solid states due to the increased *π*‐surface area resulting from the longer aromatic linkers. **37** can generate CPL in solution, indicating that the naphthalenediimide units are restricted by the rigid structure of the triangular molecules. Moreover, **37** can assemble into a uniform supramolecular helical structure via a drop‐casting strategy, resulting in the CPL signal being inverted and enhanced simultaneously. Additionally, with a tailored electron‐deficient outer surface, **37** can also assemble with achiral electron‐rich anthracene to form CT complexes. Crystal structure analysis confirms that anthracene is mounted on the outer surface of **37** through *π‐π* stacking and C‐H···*π* interactions. The co‐assemblies can generate a yellow‐green CT‐CPL.

In short, a key advantage of this assembly modes lies in its ability to induce CPL activity in achiral luminophores irrespective of their inherent chirality. This opens exciting avenues for the design and construction of CPL‐active materials. By adjusting both chiral and achiral components, a diverse array of CPL‐active materials with tunable color and handiness can be created through supramolecular assembly. These advantages stem from the crucial non‐covalent interactions between chiral and achiral moieties, which facilitate the chirality transfer from the chiral moieties to the supramolecular assemblies.

### 3.3 CPL from the assembly of achiral luminescent molecules

**FIGURE 8:**
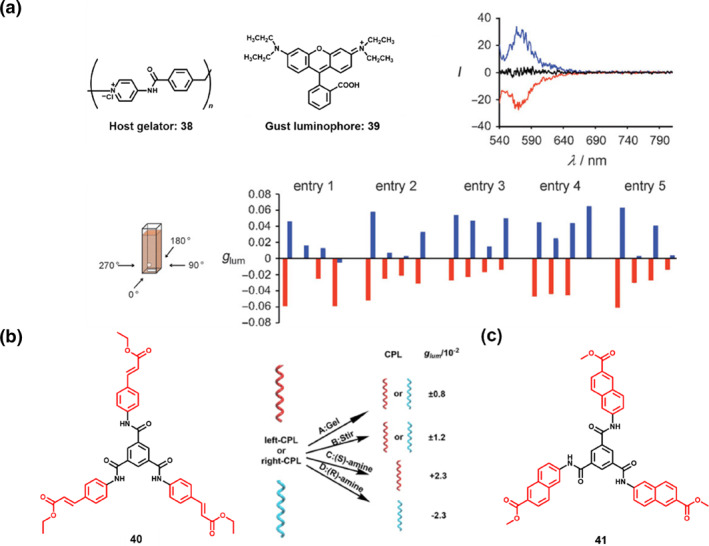
(a) CPL of **39** in a supramolecular medium under vortex flow. Molecular structures of host gelator **38** and guest luminophore **39** as well as CPL spectra prepared by CW (red), CCW (blue) stirring and without stirring (black). Statistical distributions of |*g*_lum_| values in five samples prepared by CW (red) and CCW (blue) stirring across four faces of the sample cuvette. Reproduced with permission.^[58]^ Copyright 2011, Wiley‐VCH. (b) The first circularly polarized supramolecular assembled exclusively from the achiral *C*_3_‐symmetric molecule **40**. Reproduced with permission.^[59]^ Copyright 2015, Royal Society of Chemistry (color online). (c) Molecular structure of the achiral *C*_3_‐symmetric molecule **41**. CCW, counterclockwise; CPL, circularly polarized luminescence; CW, clockwise.

In the assembly modes discussed above, chiral constituents are essential to generate CPL. However, achiral molecules can also form chiral supramolecular assemblies, enabling CPL activity through symmetry breaking mechanisms.[^11^, ^55^, ^56^, ^57^] For instance, Yamashita et al.^[58]^ first reported CPL from achiral molecules. A CPL‐active assembly can be produced by a stir‐induced vortex flow with ionic polymer **38** and achiral dye **39** (Figure 8a). This assembly mode offers several advantages: firstly, the stir‐induced vortex flow generates spatiotemporal spiral architecture on a macroscopic level; secondly, the oligomer **38** can be efficiently synthesized in a one‐pot reaction with commercially available reagents; thirdly, this study revealed the control over the positive and negative signs of CPL simply by reversing the stirring direction. CCW stirring resulted in a positive CPL signal, whereas clockwise (CW) stirring elicited a negative signal. To verify the origin of the CPL, they recorded the corresponding spectra from four faces of the cuvette. Despite slight variations in the CPL intensity, the recorded spectra consistently revealed a comparable shape and uniform sign across a series of measurements, suggesting that the physical chirality imparted by stirring is effectively transferred to **39**. Unfortunately, the CPL could only be detected during the stirring process.

Symmetry breaking can also occur in achiral molecules without any external asymmetric environmental factors. In 2015, Liu's group^[59]^ designed an achiral *C*_3_‐symmetric molecule **40** with three aromatic ethyl cinnamate groups connected to a benzene ring via amide bonds (Figure 8b). Both *π‐π* interactions between aromatic rings and hydrogen bonding from the amide groups played important roles in the supramolecular assembly of **40**. Notably, the ethyl cinnamate substituents within the **40** molecular structure possess substantial spatial volumes and inherent rigidity, creating spatial congestion that disrupts symmetry, thus inducing local symmetry breaking.^[60]^ Experimental results demonstrated that in a DMF/H_2_O mixture (v/v: 5/2), achiral molecule **40** spontaneously formed twisted supramolecular structures with CPL even without existence of any chiral additives. The Φ of this assembly is 10.5%, and the |*g*_lum_| reaches 0.8 × 10^−2^. Furthermore, the |*g*_lum_| can be further enhanced to 1.2 × 10^−2^ with mechanical stirring, which can be attributed to the stirring process that potentially increased asymmetry and amplified the CPL signals. Adding chiral amine dopants also enhanced the *g*_lum_ (|*g*_lum_| = 2.3 × 10^−2^), and the handedness of CPL signals were determined by the chirality of organic amines. This work not only uncovers the mechanism of symmetry breaking but also opens new avenues for developing CPL‐active materials from achiral molecules. To further explore the potential novel functionalities of CPL‐active assemblies based on achiral *C*_3_‐symmetric molecules, they introduced naphthyl groups by linking methyl ester naphthalene to the benzene ring via amide bonds, yielding achiral *C*_3_‐symmetric molecule **41** (Figure 8c).^[61]^ The molecule **41** exhibited abundant assembly properties, forming diverse nanostructures encompassing nanobelts, nanotwists, and nanotrumpets,^[61]^ wherein the nanotwisted structures exhibited pronounced CPL.

Although the achiral molecules can assemble into chiral nanostructures exhibiting CPL through spontaneous symmetry breaking have been reported by many researchers, these assemblies often display a preferential chiral form (one enantiomer predominates over the other). Additionally, the handedness of the CPL is still quite random in most systems from achiral molecules without any chiral additives.

**FIGURE 9:**
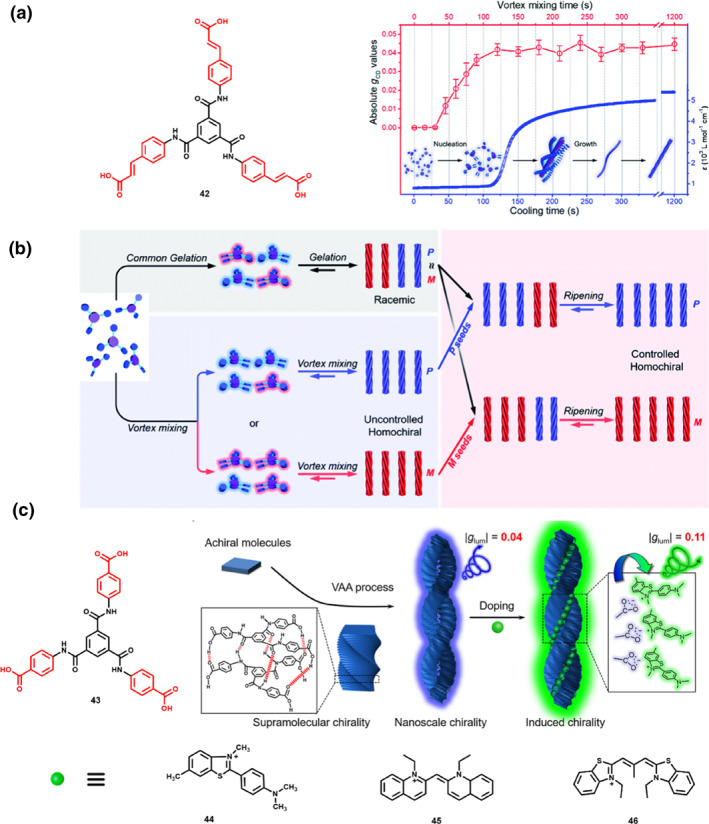
(a) Molecular structure for achiral *C*_3_‐symmetric molecule **42,** alongside the absolute *g*_CD_ values of the achiral *C*_3_‐symmetric molecule **42** assemblies prepared under varying vortex times (red curve) and the absorption data of aggregated molecules plotted as a function of cooling time (blue curve) in DMF/H_2_O (1 : 1 v/v) at 380 nm. The inset shows a possible route for this assembly. (b) Schematic illustration of vortex‐associated assembly technology was employed to achieve near‐uniform suspensions with controlled handedness. (a, b) Reproduced with permission.^[62]^ Copyright 2019, Royal Society of Chemistry. (c) Mechanically controlled and consecutively boosted circularly polarized luminescence of nanoassemblies based on achiral *C*_3_‐symmetric molecule **43**. Reproduced with permission.^[63]^ Copyright 2020, Royal Society of Chemistry.

To address these limitations, Liu et al. designed the *C*_3_‐symmetric molecule **42**, attaching three peripheral cinnamic acid moieties to a central benzene ring via amide bonds. By employing a vortex‐accompanied assembly (VAA) strategy, near‐unity homochiral assemblies with controlled handedness were obtained.^[62]^ Simply heating and cooling the achiral molecules **42** in a DMF/H_2_O mixed solvent yielded racemic gels with negligible CPL due to equal amounts of both enantiomers at small nuclei or nanohelix stages. However, introducing vortex mixing during the crucial stages of supramolecular assembly significantly promoted the formation of near‐unity homochiral assemblies with controlled handedness. Real‐time monitoring of CD intensity under various vortex mixing times revealed that vortex mixing at the nucleation stage was critical for chirality induction and amplification (Figure 9a). Under continuous vortex influence, achiral molecules initially form small nuclei with near one‐handedness growing into larger homochiral nanohelices. Notably, changing the vortex rotation direction in this case does not affect the initial handedness due to the random chiral bias, resulting in either *P* or *M* homochiral assemblies. These homochiral assemblies can act as chiral seeds disrupting the equilibrium between *P* and *M* nanohelices within racemic gels (Figure 9b). Through a ripening process, the system eventually evolves towards a near‐unity homochiral suspension with controlled handiness, which is determined by the initial chiral seeds.

**FIGURE 10:**
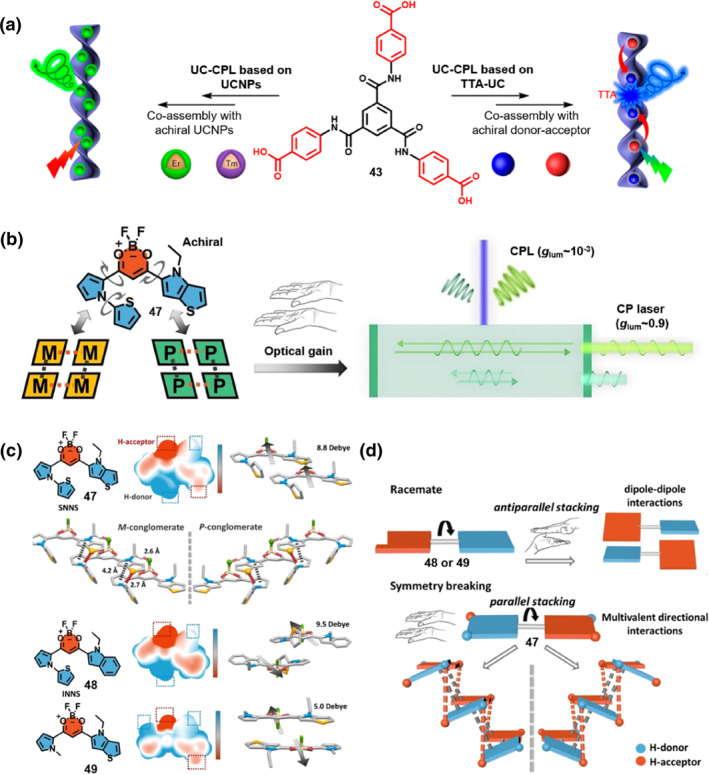
(a) Schematic illustration of upconverted CPL based on upconversion nanoparticles or triplet‐triplet annihilation photon upconversion. Reproduced with permission.^[64]^ Copyright 2021, American Chemical Society. (b) Schematic illustration of the transformation from an achiral β‐diketone boron difluoride molecule **47** to homochiral assemblies via spontaneous symmetry breaking during crystallization. The process leads to the generation of CPL with a *g*_lum_ of approximately 10^−3^ due to optical gain. Moreover, these assemblies can further enable the emission of CP laser with a significantly higher *g*_lum_ value of around 0.9. (c) Molecular structures and surface electrostatic potential of three β‐diketone boron difluoride molecules (**47–49**), parallel packing orientation of **47**, antiparallel packing orientation of 48 and 49. (d) Schematic illustration of antiparallel packing of **48** or **49**, and parallel molecular packing of **49**, which is composed of electron‐donating (D) and accepting (A) moieties. (b–d) Reproduced with permission.^[65]^ Copyright 2023, Wiley‐VCH. CPL, circularly polarized luminescence.

Although the VAA strategy was successfully employed to obtain near‐unity homochiral assemblies with controlled handedness, sufficient CPL activity is still not attained. To address this, they adopted a novel approach to investigate mechanically controlled and consecutively enhanced CPL in nanoassemblies (Figure 9c).^[63]^ They substituted benzoic acid for cinnamic esters to synthesize *C*_3_‐symmetric molecule **43**, which has been widely reported in supramolecular gel systems. The VAA approach significantly enhances chiral bias in this system (with a *g*_lum_ value reaching 4.35 × 10^−2^). Moreover, the resultant assemblies could serve as chiral seeds, which determined the handedness of CPL in the following VAA strategy. Furthermore, they explored the assembly of these chiral‐controlled assemblies with various achiral dyes (**44–46**). Among these achiral dyes, **44** is a typical fluorophore with a positive charge, and its FL intensity can increase remarkably once the freely rotating aromatic rings are fixed. **44** can be assembled into the nanoassemblies of **43** through the electrostatic interaction. The excitation energy of chiral **43** can be further transferred to another achiral dye **44**, resulting in a larger *g*_lum_ of 0.11. By integrating symmetry breaking, VAA strategy, and energy transfer mechanisms, this research opens new pathways for offering sufficient chiral amplification and designing high‐performance CPL‐active materials based on achiral molecules. In 2021, they further employed the achiral *C*_*3*_‐symmetric molecule **43** to fabricate upconverted (UP)‐CPL‐active materials (Figure 10a).^[64]^ They selected two different photon upconversion systems, namely the triplet‐triplet annihilation photon upconversion (TTA‐UC) donor/acceptor pairs and inorganic lanthanide upconversion nanoparticles (UCNPs). Upon the assembly of these two systems with the chiral nanohelix fabricated from molecule **43**, hybrid nanohelices were generated, and this process led to the induction of UP‐CPL activity.

In addition to *C*_3_‐symmetric molecules as building blocks, achiral molecules composed of electron‐donating (D) and accepting (A) moieties have emerged in this field. Yang et al. took D‐A type achiral molecules as models, achieved chiral induction through the regulation of weak intermolecular interactions, and proposed a theory for the spontaneous symmetry breaking of D‐A type achiral molecules (Figure 10b).^[65]^ They designed three β‐diketone boron difluoride molecules (**47–49**) with different hydrogen‐bonding sites and flexible conformations. Their CD and CPL spectra in solution, and chiral high‐performance liquid chromatography analysis indicated that all three molecules were achiral in solution. Single‐crystal XRD results showed that only **47** underwent symmetry breaking during the crystallization process, forming homochiral crystals. By comparing the packing structures of three β‐diketone boron difluoride molecules, they found that the symmetry of molecular assembly was broken by multiple non‐covalent interactions, including hydrogen bonds (Figure 10c). The directionality of hydrogen bonds provides orientation for packing, overriding the original dipole‐dipole interactions of **47**, promotes the parallel packing, and realizes spontaneous symmetry breaking (Figure 10d). Additionally, **47** exhibits well‐defined hierarchical assembly in the crystal. The molecular chirality is transferred to a higher‐order helical structure during the hierarchical assembly process, forming supramolecular chirality. Unlike traditional ACQ dyes, **47** shows bright green FL emission and significant optical activity in the crystalline state, with both the *g*_abs_ and the *g*_lum_ on the order of 10^−3^. Moreover, the homochiral structure formed by crystallization exhibits excellent circularly polarized lasing properties, providing new ideas for the design of chiral laser materials.

In brief, the assembly of achiral components provides a new perspective for investigating CPL‐active materials. Nonetheless, current studies reveal that only a limited number of organic emitters with specific molecular architectures can show symmetry breaking during supramolecular assembly. It now necessitates sophisticated advancements in molecular design and assembly process regulation to construct more CPL‐active materials.

## 4 APPLICATIONS OF CPL‐ACTIVE MATERIALS

Intensive research by chemists in the field of CPL has greatly advanced the scope and potential of CPL‐active materials. Beyond its pivotal role in biology‐related fields such as in natural signaling for predation and mating,[^66^, ^67^, ^68^] its applications in chiral optoelectronics have opened new technological possibilities. This section delves into the applications of CPL in circularly polarized organic light‐emitting diodes (CP‐OLEDs) and sensing technologies.

**FIGURE 11:**
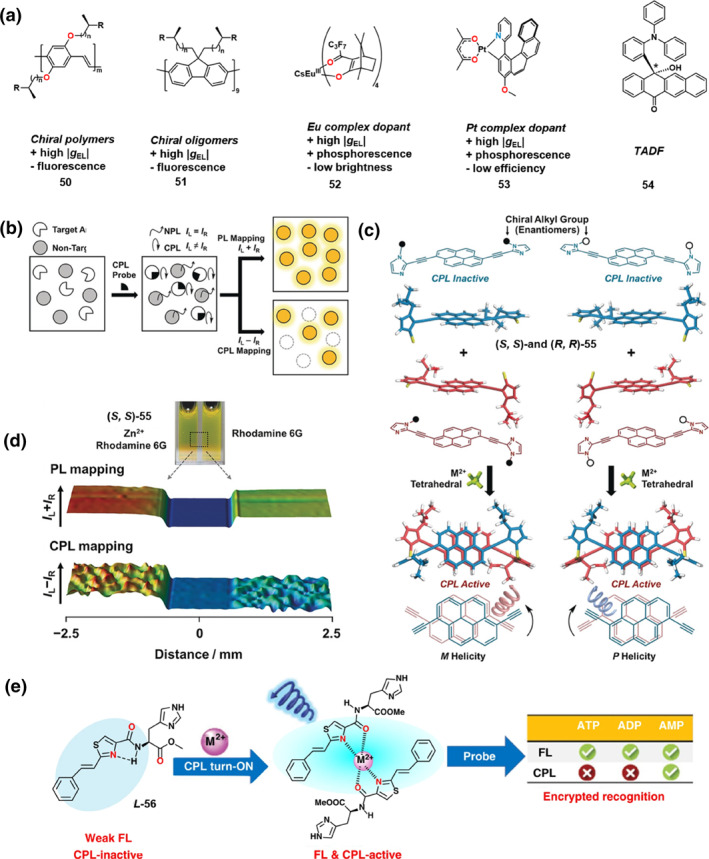
(a) Typical molecular structures of CPL‐active materials for circularly polarized organic light‐emitting diodes. (b) Illustration of sensor method using the CPL signal as detection output for object identification (c) Molecular structures of (*S*, *S*)‐ and (*R*, *R*)‐**55** and illustration of using the CPL signal for object identification. (d) Images showing visible‐emission, PL and CPL mapping (detection at 550 nm) of a sample containing Rhodamine 6G (right) compared to another sample with (*S*, *S*)‐**55**, Zn^2+^ and Rhodamine 6G (left). The dashed square in the photograph indicates the PL and CPL mapping area. (b–d) Reproduced with permission.^[73]^ Copyright 2018, Wiley‐VCH. (e) Molecular structure of ***L‐*56** and encrypted selective recognition of adenosine monophosphate. Reproduced with permission.^[74]^ Copyright 2020, Wiley‐VCH. CPL, circularly polarized luminescence.

To achieve high image contrast in OLEDs, circular polarizers are employed to minimize ambient reflectivity. However, this method comes with a trade‐off, as it reduces the emitted light by half, resulting in significant brightness loss and increased energy consumption. CP‐OLEDs, emitting circularly polarized light with the same handedness as the polarizer can prevent this loss and avoid unnecessary power consumption.[^60^, ^61^, ^62^, ^63^, ^64^, ^65^, ^66^, ^67^, ^68^, ^69^, ^70^] Since the first CP‐OLED based on chiral polymer **50** was reported in 1997,^[71]^ various designs have emerged. In 2003, Chen's group^[72]^ achieved a *g*_EL_ of 0.35 using oligomers **51** based on a polyfluorene backbone with different chiral alkyl side chains (Figure 11a). However, the current efficiency (CE_max_) was limited to 0.94 cd·A^−1^. Subsequently, the introduction of chiral Eu(III) enantiomers and Pt(II) complexes further improved the performance of CP‐OLEDs. In 2015, Bari's group^[1]^ reported CP‐OLEDs based on chiral Eu(III) enantiomers **52** (Figure 11a). While the |*g*_EL_| value of these devices was enhanced to 0.75, their efficiency was still relatively low. The CE_max_ was 5 × 10^−3^ cd·A^−1^, and the maximum external quantum efficiency (EQE_max_) was 4.2 × 10^−3^%. To achieve high device efficiency, Fuchter et al.^[75]^ developed a phosphorescent CP‐OLED utilizing Pt(II) complexes **53** (Figure 11a), which exhibited a high CE_max_ of 0.52 cd·A^−1^, and a *g*_EL_ value of −0.38. Although progress has been made in device efficiency using chiral polymers, oligomers, Eu(III), and Pt(II) complexes, further improvements in brightness and efficiency are essential. In this regard, TADF materials emerged as the third‐generation emitters. Their efficient upconversion process can theoretically achieve an internal quantum efficiency of 100%, and the device efficiency is comparable to that of phosphorescent emitters or even exceeds 30%. Imagawa et al.^[76]^ first reported a CPL‐active TADF molecule **54** (Figure 11a) with a *g*_lum_ of 10^−3^. However, devices based on its enantiomers have not been realized, and their CPEL properties have not been detected. Despite the limitations, this strategy undoubtedly offers a new perspective to explore CP‐OLEDs. Currently, the |*g*_EL_| values of most CP‐OLEDs are in the range of 10^−4^ to 10^−3^, significantly lower than the |*g*| values of 0.01–1 achievable by non‐emissive liquid crystals, which hinders the application and widespread adoption of CP‐OLEDs.

Additionally, CPL‐active materials also show promising potential in chemical and biological sensing. Compared to other optical sensing technologies, CPL‐based sensing can effectively eliminate background FL and unpolarized light interference, leading to enhanced sensitivity and resolution. As shown in Figure 11b, Yuasa's group^[73]^ developed a chiral luminescent probe **55** for object identification. When coordinated with metal ions that prefer tetrahedral coordination, such as Zn^2+^, these probe molecules can assemble into complexes with well‐defined *P*‐ or *M*‐type helical structures. These structures enhanced exciton interactions and endowed the complexes with prominent CPL signals, in sharp contrast with the non‐polarized light exhibited by non‐recognition substances such as Rhodamine 6G (Figure 11c). Samples containing the chiral probe **55**, Zn^2+^, and Rhodamine 6G showed a photoluminescence spectrum similar to that of Rhodamine 6G. However, CPL mapping distinctly revealed a clear difference: intense CPL signals were detected in samples containing Zn^2+^ and the chiral probe, whereas no CPL signal was observed in Rhodamine 6G (Figure 11d).^[73]^ Similarly, a highly sensitive sensing of Al^3+^ was developed by leveraging the coordination chemistry between Schiff bases and metal ions.^[77]^ Furthermore, CPL sensing technology has garnered widespread application for the selective recognition of bioactive molecules.[^78^, ^79^, ^80^] Recently, Zhao et al.^[74]^ designed a metal complex comprising a histidine derivative (***L*‐56**) and Mg^2+^ as a CPL probe, enabling the selective recognition of adenosine monophosphate (AMP) among the three types of adenosine phosphates (ATP, ADP, and AMP). While FL signals cannot distinguish between these phosphates (Figure 11e), CPL measurements revealed that ATP and ADP caused quenching of the CPL signal, whereas AMP preserved CPL activity, thus enabling specific recognition of AMP. This work introduces a CPL‐based encrypted detection approach for the selective identification of AMP, advancing the application of CPL probes in sensing of chiral bioactive molecules.

While the progress in CPL‐active materials is promising, their applications are still in their infancy. Current |*g*_lum_| values are still too low, and higher |*g*_lum_| is essential for advancing technologies. Continued innovation in molecular design and assembly techniques will be crucial to overcome these challenges and unlock the full potential of CPL‐active materials.

## 5 CONCLUSION AND PERSPECTIVE

This review begins by elucidating the fundamental concepts of CPL, subsequently focusing on recent processes made in CPL‐active supramolecular assemblies based on small molecules. In general, a conventional CPL‐active material comprises two principal components: chiral moieties and luminescent moieties. Three typical assembly modes have been widely applied to generate CPL: (1) the assembly of chiral luminescent molecules; (2) the assembly of chiral molecules and achiral luminescent molecules; (3) the assembly of achiral luminescent molecules. Furthermore, these assemblies show their potential in applications such as CP‐OLED, chemical and biological sensing, and so on.

Although considerable progress has been made in CPL‐active supramolecular assemblies based on small molecules, there still remain several challenges. Firstly, there is an urgent need for a thorough exploration of chirality transfer mechanisms from chiral sources to the chiral assemblies. Moreover, these assemblies often exhibit poor stability. The formation of CPL‐active assemblies necessitates precise and ordered arrangements of small molecules. However, achieving and maintaining such arrangements is challenging, primarily because the supramolecular assembly process is sensitive to external factors such as solvent, temperature, and concentration. Thirdly, developing advanced CPL systems that exhibit both high Φ and |*g*_lum_| is crucial, as the balance between these two parameters continues to pose significant hurdles. Despite the continuous development of new approaches to enhance both Φ and |*g*_lum_|, the currently attainable levels of both Φ and |*g*_lum_| remain insufficient to satisfy the demands of practical applications. Advancements in the areas of ACQ and AIE have greatly facilitated the design of chiral materials showing CPL activity. Fourth, macrocycle‐based CPL systems are constructed. Macrocycles, compared with non‐cyclic building blocks, can display diverse assembly behaviors due to their unique cavity structure. However, as research on macrocycle‐based CPL systems is still in its infancy, there is enormous room for optimizing macrocycle assemblies. Ultimately, limited applications have been explored based on these assemblies. Therefore, significant further exploration and effort are necessary in this area. We hope to stimulate the interest among scientists and researchers, encouraging them to intensify their endeavors toward unraveling novel findings in chiral supramolecular assemblies and advancing the design and utilization of CPL‐active materials.

## CONFLICT OF INTEREST STATEMENT

The authors declare no conflicts of interest.

## Data Availability

Data sharing is not applicable to this article as no new data were created or analyzed in this study.
